# Associations of *MTHFR* C677T and *MTRR* A66G Gene Polymorphisms with Metabolic Syndrome: A Case-Control Study in Northern China

**DOI:** 10.3390/ijms151221687

**Published:** 2014-11-25

**Authors:** Boyi Yang, Shujun Fan, Xueyuan Zhi, Da Wang, Yongfang Li, Yinuo Wang, Yanxun Wang, Jian Wei, Quanmei Zheng, Guifan Sun

**Affiliations:** 1 Environment and Non-Communicable Disease Research Center, School of Public Health, China Medical University, Shenyang 110013, China; E-Mails: boyiyangcmu@163.com (B.Y.); fanfan0721ykl@163.com (S.F.); zhixy90smile@126.com (X.Z.); 472594283@163.com (D.W.); liyongfang17@163.com (Y.L.); qmzheng@mail.cmu.edu.cn (Q.Z.); 2 Division of Molecular Preventive Medicine, Shanghai Institute of Targeted Therapy and Molecular Medicine, Shanghai 200433, China; E-Mails: 13817895706@163.com (Y.W.); wangyanxun@genechina.com (Y.W.); 3 Brain Disease Center, Tianjin Dagang Oil Field General Hospital, Tianjin 300280, China; E-Mail: weijiantianjin@163.com

**Keywords:** *MTHFR*, *MTRR*, polymorphism, metabolic syndrome, China

## Abstract

Prior evidence indicates that homocysteine plays a role in the development of metabolic syndrome (MetS). Methylenetetrahydrofolate reductase (*MTHFR*) C677T and methionine synthase reductase (*MTRR*) A66G polymorphisms are common genetic determinants of homocysteine levels. To investigate the associations of the *MTHFR* C677T and *MTRR* A66G polymorphisms with MetS, 692 Chinese Han subjects with MetS and 878 controls were recruited. The component traits of MetS and the *MTHFR* C677T and *MTRR* A66G genotypes were determined. A significant association was observed between the *MTHFR* 677T allele and increased risk of MetS, high fasting blood glucose, high waist circumference, and increasing number of MetS components. The *MTRR* A66G polymorphism was associated with an increased risk of MetS when combined with the *MTHFR* 677TT genotype, although there was no association found between MetS and *MTRR* A66G alone. Furthermore, the *MTRR* 66GG genotype was associated with high fasting blood glucose and triglycerides. Our data suggest that the *MTHFR* 677T allele may contribute to an increased risk of MetS in the northern Chinese Han population. The *MTRR* A66G polymorphism is not associated with MetS. However, it may exacerbate the effect of the *MTHFR* C677T variant alone. Further large prospective population-based studies are required to confirm our findings.

## 1. Introduction

Metabolic syndrome (MetS) is characterized through a cluster of various metabolic abnormalities, including central obesity, dyslipidemia, elevated blood pressure (BP) and high glucose concentrations. Available evidence shows that the prevalence of MetS is rapidly increasing worldwide [1,2]. In China, the prevalence has been estimated as 13.7% in men and 17.8% in women, and shows geographical and age variations [3]. MetS and its individual components have been reported to be associated with increased risk of diabetes and cardiovascular diseases, as well as with high all-cause mortality [4,5,6]. The etiology of MetS is complicated because many genetic and environmental factors may be involved [6]. Homocysteine (Hcy) is a sulfur-containing amino acid derived from methionine metabolism. Several studies have linked hyperhomocysteinemia (HHcy) to MetS and its individual components [7,8,9,10,11,12], and it has even been described as an integral component of MetS in rats [13]. Potential mechanisms linking HHcy with MetS and its components include promoting endothelial dysfunction, inducing insulin resistance, and influencing DNA methylation status [10,14,15,16,17]. Because of the relationship between Hcy and MetS, factors influencing the plasma Hcy levels may potentially relate to MetS.

Plasma Hcy levels are controlled and modulated by multiple genetic and environmental factors and their interactions. Among these factors, methylenetetrahydrofolate reductase (*MTHFR*) C677T and methionine synthase reductase (*MTRR*) A66G polymorphisms are the major genetic causes. *MTHFR,* which catalyzes the conversion of 5,10-methylenetetrahydrofolate to 5-methyltetrahydrofolate, is an enzyme necessary for the Hcy metabolic pathway and has been associated with methylation of genomic DNA. Polymorphism of the *MTHFR* gene at position 677 from C to T decreases its enzyme activity, leading to accumulation of Hcy especially under conditions of low dietary folate [18]. Previous studies also showed that the *MTHFR* 677T allele could cause DNA hypomethylation, which has been associated with MetS and its components [15,19]. *MTRR* is another important enzyme involved in Hcy metabolism, which is responsible for the remethylation of Hcy to methionine via a vitamin B_12_ dependent reaction [20]. The most common polymorphism in the *MTRR* gene is the substitution of A for G at position 66, which adversely influences the enzyme activity and thus is considered as a genetic risk factor for HHcy [20]. The *MTRR* A66G polymorphism may also induce DNA hypomethylation by regulation of Hcy levels [14]. Additionally, some studies suggested additive or synergistic effects of the *MTHFR* C677T and *MTRR* A66G polymorphisms on plasma Hcy levels [21].

Previous epidemiological studies have indicated that carriers of the *MTHFR* 677T allele or 677TT genotype were at significantly increased risk of developing hypertension [22], type 2 diabetes [23], dyslipidemia [24] and obesity [25], which are all important factors in the diagnosis of MetS. With respect to the *MTRR* A66G polymorphism, its effect on MetS is not as well studied as that of the *MTHFR* C677T polymorphism and, to date, no study has directly explored the relationship between the *MTRR* A66G polymorphism and MetS. Additionally, some studies have investigated the effect of the *MTHFR* C677T polymorphism on MetS in schizophrenia [26,27], type 2 diabetes [28], stroke [29] and colorectal cancer patients [30], however, few studies have examined the risk of MetS in the general population without severe disease. In China, due to the changing life styles and dietary habits, the rates of MetS and its related diseases such as stroke, coronary heart disease, hypertension and diabetes, are markedly increasing, especially in northern areas [31]. Previous studies reported that residents of northern China had higher plasma Hcy concentrations than those living in southern China [32]. Our group also has observed high frequencies of the *MTHFR* 677TT genotype and 677T allele among Chinese northerners [33]. Based on the above considerations, we hypothesized that the *MTHFR* C677T and *MTRR* A66G polymorphisms, either independently or in combination, might be associated with MetS in a northern Chinese Han population. We therefore conducted a case-control study to investigate the above hypothesis in a population living in Tianjin, which is a municipality located in northern China.

## 2. Results

### 2.1. Demographic and Clinical Characteristics of Study Subjects

Table 1 summarizes the demographic and clinical characteristics of the MetS patients and controls. The distribution of sex and age was similar between the two groups. However, significant differences existed between the two groups in body mass index (BMI), waist circumference (WC), systolic blood pressure (SBP), diastolic blood pressure (DBP), fasting blood glucose (FBG), triglyceride (TG), total cholesterol (TC), high density lipoprotein cholesterol (HDL-C) and low density lipoprotein cholesterol (LDL-C) levels.

**Table 1:** Demographic and clinical characteristics of study subjects.

Variables	Patients	Controls	*p* Value
Gender (M/F)	541/151	659/219	0.1478
Age (year)	48.82 ± 10.10	46.54 ± 9.93	0.0621
BMI (kg/m^2^)	27.37 ± 3.55	24.14 ± 3.14	<0.0001
WC (cm)	94.35 ± 8.46	84.52 ± 9.08	<0.0001
SBP (mmHg)	141.40 ± 17.88	125.30 ± 17.11	<0.0001
DBP (mmHg)	90.05 ± 12.02	79.74 ± 11.81	<0.0001
FBG (mmol/L)	5.79 ± 1.45	5.02 ± 0.72	<0.0001
TG (mmol/L)	2.12 ± 1.79	1.01 ± 0.60	<0.0001
TC (mmol/L)	5.13 ± 1.04	4.91 ± 0.91	<0.0001
HDL-C (mmol/L)	1.03 ± 0.25	1.26 ± 0.32	<0.0001
LDL-C (mmol/L)	2.82 ± 0.98	2.69 ± 0.88	0.0081

Abbreviations: M, male; F, female; BMI, body mass index; WC, waist circumference; SBP; systolic blood pressure; DBP, diastolic blood pressure; FBG, fasting blood glucose; TG, triglycerides; TC, total cholesterol; HDL-C, high density lipoprotein cholesterol; LDL-C, low density lipoprotein cholesterol.

### 2.2. Genotypic and Allelic Frequencies

Genotypic and allelic frequencies for the *MTHFR* C677T and *MTRR* A66G polymorphisms are presented in Table 2 and Table 3, respectively. Genotype distributions of the two polymorphisms were consistent with Hardy–Weinberg equilibrium in both patients (*p* = 0.7610) and controls (*p* = 0.6383)*.* No significant differences in the prevalence of the *MTHFR* C677T and *MTRR* A66G polymorphisms were found between males and females (*p* = 0.3603 and 0.5202, respectively).

For the *MTHFR* C677T polymorphism, significant differences were found in the genotypic (*p* = 0.0082) and allelic (*p* = 0.0091) frequencies between the patients and controls. For the *MTRR* A66G polymorphism, no statistically significant differences were detected between the two groups in either the genotypic frequencies (*p* = 0.2704) or allelic frequencies (*p* = 0.4012).

**Table 2:** Association of methylenetetrahydrofolate reductase (*MTHFR*) C677T polymorphism with metabolic syndrome (MetS) risk.

Genetic Model	Patients (*n* = 692)	Controls (*n* = 878)	Crude OR (95% CI)	*p* Value	Adjusted OR ^a^ (95% CI)	*p* Value
Codominant						
CC	129 (18.6)	202 (23.0)	1.00		1.00	
CT	335 (48.4)	431 (49.1)	1.22 (0.94–1.58)	0.1432	1.24 (0.95–1.62)	0.1129
TT	228 (32.9)	245 (27.9)	1.46 (1.10–1.94)	0.0097	1.48 (1.11–1.98)	0.0076
Dominant						
CC	129 (18.6)	202 (23.0)	1.00		1.00	
CT+TT	563 (81.4)	676 (77.0)	1.30 (1.02–1.67)	0.0355	1.33 (1.03–1.71)	0.0266
Recessive						
CC+CT	464 (67.1)	633 (72.1)	1.00		1.00	
TT	228 (32.9)	245 (27.9)	1.27 (1.02–1.58)	0.0306	1.27 (1.02–1.59)	0.0302
Allelic						
C	593 (42.8)	835 (47.6)	1.00		1.00	
T	791 (57.2)	921 (52.4)	1.21 (1.05–1.39)	0.0086	1.22 (1.10–1.34)	0.0002

Abbreviations: *MTHFR*, methylenetetrahydrofolate reductase; OR, odds ratio; CI, confidence interval; MetS, metabolic syndrome; ^a^ Adjusted for age and sex.

**Table 3:** Association of methionine synthase reductase (*MTRR*) A66G polymorphism with MetS risk.

Genetic Model	Patients (*n* = 692)	Controls (*n* = 878)	Crude OR (95% CI)	*p* Value	Adjusted OR ^a^ (95% CI)	*p* Value
Codominant						
AA	391 (56.5)	501 (57.1)	1.00		1.00	
AG	245 (35.4)	324 (36.9)	0.97 (0.78–1.20)	0.7705	0.99 (0.80–1.23)	0.9444
GG	56 (8.1)	53 (6.0)	1.35 (0.91–2.02)	0.1359	1.37 (0.92–2.05)	0.1270
Dominant						
AA	391 (56.5)	501 (57.1)	1.00		1.00	
AG + GG	301 (43.5)	377 (42.9)	1.02 (0.84–1.25)	0.8244	1.05 (0.85–1.28)	0.6683
Recessive							
AA + AG	636 (91.9)	825 (94.0)	1.00		1.00		
GG	56 (8.1)	53 (6.0)	1.37 (0.93–2.02)	0.1127	1.37 (0.93–2.04)	0.1153	
Allelic							
A	1027 (74.2)	1326 (75.5)	1.00		1.00		
G	357 (25.8)	430 (24.5)	1.07 (0.91–1.26)	0.4014	1.08 (0.97–1.22)	0.1639	

Abbreviations: *MTRR*, methionine synthase reductase; OR, odds ratio; CI, confidence interval; MetS, metabolic syndrome; ^a^ Adjusted for age and sex.

### 2.3. Association between Gene Polymorphisms and Metabolic Syndrome (MetS)

Codominant, dominant, recessive, and allelic genetic models were applied to test the associations of the *MTHFR* C677T and *MTRR* A66G polymorphisms with MetS risk. The *MTHFR* C677T polymorphism was associated with an increased risk of MetS under the homozygous codominant model (odds ratio (OR) = 1.46, 95% confidence interval (CI) = 1.10–1.94, *p* = 0.0097), dominant model (OR = 1.30, 95% CI = 1.02–1.67, *p* = 0.0355), recessive model (OR = 1.27, 95% CI = 1.02–1.58, *p* = 0.0306) and allelic model (OR = 1.21, 95% CI = 1.05–1.39, *p* = 0.0086) (Table 2). The associations were almost unchanged after adjusting for age and gender (Table 2). With respect to the *MTRR* A66G polymorphism, no significant association was found under any of the tested genetic models (Table 3).

We then analyzed the association between combined *MTHFR* C677T and *MTRR* A66G genotypes and the risk of MetS. By using combined CC/AA genotype as a reference, TT/GG genotype showed a significant association with MetS (OR = 2.10, 95% CI = 1.07–4.12, *p*= 0.0307) (Table 4). In addition, both TT/AA and TT/AG genotypes showed borderline associations with MetS (OR = 1.46, 95% CI = 0.99–2.16, *p* = 0.0539, and OR = 1.52, 95% CI = 0.98–2.33, *p* = 0.0597, respectively) (Table 4).

**Table 4:** Interaction of *MTHFR* C677T and *MTRR* A66G polymorphisms on MetS risk.

Combined Genotype	Patients (*n* = 692)	Controls (*n* = 878)	Crude OR (95% CI)	*p* Value	Adjusted OR ^a^ (95% CI)	*p* Value
CC/AA	71 (10.3)	113 (12.9)	1.00		1.00	
CC/AG	46 (6.6)	78 (8.9)	0.94 (0.59–1.50)	0.7916	0.98 (0.61–1.58)	0.9442
CC/GG	12 (1.7)	11 (1.3)	1.74 (0.73–4.15)	0.2141	1.73 (0.72–4.18)	0.2198
CT/AA	195 (28.2)	248 (28.2)	1.25 (0.88–1.78)	0.2106	1.28 (0.90–1.83)	0.1677
CT/AG	121 (17.5)	160 (18.2)	1.20 (0.82–1.76)	0.3382	1.26 (0.86–1.85)	0.2398
CT/GG	19 (2.7)	23 (2.6)	1.32 (0.67–2.59)	0.4277	1.43 (0.72–2.83)	0.3116
TT/AA	125 (18.1)	140 (15.9)	1.42 (0.97–2.08)	0.0717	1.46 (0.99–2.16)	0.0539
TT/AG	78 (11.3)	86 (9.8)	1.44 (0.94–2.21)	0.0917	1.52 (0.98–2.33)	0.0597
TT/GG	25 (3.6)	19 (2.2)	2.09 (1.08–4.08)	0.0297	2.10 (1.07–4.12)	0.0307

Abbreviations: *MTHFR*, methylenetetrahydrofolate reductase; *MTRR*, methionine synthase reductase; OR, odds ratio; CI, confidence interval; MetS, metabolic syndrome; ^a^ Adjusted for age and sex.

### 2.4. Association between Gene Polymorphisms and Individual Components of MetS

We also investigated the associations of individual components of MetS with the *MTHFR* C677T and *MTRR* A66G polymorphisms. Multivariate logistic regression analysis showed that the *MTHFR* C677T polymorphism increased the risk of high FBG in the homozygous codominant (OR = 1.83, 95% CI = 1.30–2.57, *p* = 0.0005), heterozygous codominant (OR = 1.79, 95% CI = 1.30–2.45, *p* = 0.0003), dominant (OR = 1.80, 95% CI = 1.34–2.44, *p* = 0.0001) and allelic (OR = 1.28, 95% CI = 1.10–1.50, *p* = 0.0020) models, and increased the risk of high WC in the recessive model (OR = 1.25, 95% CI = 1.00–1.57, *p* = 0.0413). No significant association was observed between the *MTHFR* C677T polymorphism and high BP, high TG, and low HDL-C under any of the tested genetic models (Table 5). In addition, we found positive associations between having more MetS components and the *MTHFR* C677T polymorphism (Table 6).

**Table 5:** Association of *MTHFR* C677T and *MTRR* A66G polymorphisms with individual components of MetS.

Age and Sex Adjusted OR (95% CI)					
Genetic Model	High BP	High FBG	High TG	Low HDL-C	High WC
*MTHFR* C677T polymorphism					
Homozygous codominant	1.25 (0.94–1.67)	1.83 (1.30–2.57)	1.20 (0.87–1.67)	1.02 (0.77–1.36)	1.33 (0.99–1.79)
Heterozygous codominant	1.07 (0.82 –1.39)	1.79 (1.30–2.45)	1.29 (0.96–1.74)	0.94 (0.72–1.22)	1.09 (0.84–1.43)
Dominant	1.13 (0.88–1.45)	1.80 (1.34–2.44)	1.26 (0.95–1.67)	0.97 (0.76–1.24)	1.18 (0.92–1.52)
Recessive	1.20 (0.96–1.49)	1.20 (0.95–1.53)	1.01 (0.79–1.28)	1.07 (0.86–1.33)	1.25 (1.00–1.57)
Allelic	1.10 (0.96–1.27)	1.28 (1.10–1.50)	1.06 (0.91–1.24)	1.04 (0.90–1.19)	1.12 (0.97–1.29)
*MTRR* A66G polymorphism					
Homozygous codominant	1.17 (0.78–1.75)	1.86 (1.22–2.82)	1.61 (1.06–2.46)	0.75 (0.50–1.14)	0.91 (0.60–1.38)
Heterozygous codominant	1.11 (0.90–1.38)	0.97 (0.77–1.24)	0.90 (0.70–1.14)	0.86 (0.69–1.06)	0.91 (0.73–1.14)
Dominant	1.12 (0.92–1.38)	1.09 (0.87–1.37)	0.99 (0.79–1.25)	0.84 (0.69–1.03)	0.91 (0.74–1.12)
Recessive	1.12 (0.75–1.67)	1.88 (1.25–2.82)	1.68 (1.12–2.54)	0.80 (0.53–1.19)	0.95 (0.63–1.42)
Allelic	1.07 (0.91–1.26)	1.16 (0.97–1.39)	1.08 (0.90–1.29)	0.88 (0.74–1.03)	0.91 (0.78–1.07)

Abbreviations: *MTHFR*, methylenetetrahydrofolate reductase; *MTRR*, methionine synthase reductase; OR, odds ratio; CI, confidence interval; BP, blood pressure; FBG, fasting blood pressure; TG, triglycerides; HDL-C, high density lipoprotein cholesterol; WC, waist circumference.

**Table 6:** Adjusted odds ratios for *MTHFR* C677T and *MTRR* A66G polymorphisms according to the number of components of MetS.

Age and Sex Adjusted OR (95% CI)				
Genetic Model	≥1 Components	≥2 Components	≥3 Components	≥4 Components
*MTHFR* C677T polymorphism				
Homozygous codominant	1.30 (0.87–1.95)	1.46 (1.08–1.97)	1.50 (1.12–2.02)	1.68 (1.10–2.27)
Heterozygous codominant	1.05 (0.74–1.50)	1.26 (0.96–1.65)	1.26 (0.96–1.66)	1.52 (1.02–2.27)
Dominant	1.14 (0.81–1.59)	1.33 (1.03–1.71)	1.35 (1.04–1.75)	1.58 (1.08–2.31)
Recessive	1.26 (0.92–1.73)	1.24 (0.99–1.56)	1.28 (1.02–1.60)	1.24 (0.92–1.67)
Allelic	1.13 (0.93–1.38)	1.17 (1.01–1.35)	1.20 (1.04–1.39)	1.24 (1.01–1.52)
*MTRR* A66G polymorphism				
Homozygous codominant	1.43 (0.76–2.70)	1.43 (0.92–2.23)	1.42 (0.95–2.13)	0.80 (0.44–1.44)
Heterozygous codominant	0.95 (0.71–1.28)	0.93 (0.75–1.17)	0.92 (0.73–1.14)	0.86 (0.64–1.17)
Dominant	1.01 (0.76–1.34)	1.00 (0.81–1.23)	0.99 (0.80–1.21)	0.85 (0.64–1.14)
Recessive	1.46 (0.79–2.72)	1.47 (0.95–2.27)	1.47 (0.99–2.19)	0.84 (0.47–1.51)
Allelic	1.05 (0.84–1.32)	1.03 (0.88–1.22)	1.04 (0.88–1.23)	0.86 (0.68–1.09)

Abbreviations: *MTHFR*, methylenetetrahydrofolate reductase; *MTRR*, methionine synthase reductase; OR, odds ratio; CI, confidence interval.

For the *MTRR* A66G polymorphism, significant associations were found between the polymorphism and high FBG in the homozygous codominant (OR = 1.86, 95% CI = 1.22–2.82, *p* = 0.0036) and recessive (OR = 1.88, 95% CI = 1.25–2.82, *p* = 0.0024) models. The polymorphism was also associated with increased risk of high TG in the homozygous codominant (OR = 1.61, 95% CI = 1.06–2.46, *p* = 0.0260) and recessive (OR = 1.68, 95% CI = 1.12–2.54, *p* = 0.0130) models. No significant association was observed between the *MTRR* A66G polymorphism and the remaining three components in any of the tested genetic models (Table 5).

When evaluating the gene-gene interaction, the combined CC/GG, CT/AA, CT/AG, CT/GG, TT/AA, TT/AG and TT/GG genotypes increased the risk of high FBG with an OR of 3.08 (95% CI = 1.21–7.82, *p* = 0.0179), 2.08 (95% CI = 1.35–3.21, *p* = 0.0009), 1.94 (95% CI = 1.22–3.09, *p* = 0.0050), 2.90 (95% CI = 1.37–6.12, *p* = 0.0054), 1.98 (95% CI = 1.24–3.17, *p* = 0.0040), 1.95 (95% CI = 1.16–3.26, *p* = 0.0112), and 3.95 (95% CI = 1.93–8.05, *p* = 0.0002), respectively. And the combined CT/GG genotype was associated with increased risk of high TG (OR = 2.65, 95% CI = 1.30–5.41, *p* = 0.0072) (Table 7). In addition, the combined TT/GG genotype was associated with a 2.52-fold (95% CI = 1.15–5.51, *p* = 0.0208) increased risk of having ≥2 MetS components and a 1.98-fold (95% CI = 1.01–3.89, *p* = 0.0470) increased risk of having ≥3 MetS components. The CT/AA and TT/AA genotypes were associated with a 2.02-fold (95% = 1.15–3.54, *p* = 0.0144) and a 2.49-fold (95% CI = 1.38–4.49, *p* = 0.0024) increased risk of having ≥4 MetS components, respectively (Table 8).

**Table 7:** Association of combined *MTHFR* C677T and *MTRR* A66G genotypes with MetS.

Age and Sex Adjusted OR with 95% CI					
Combined Genotype	High BP	High FBG	High TG	Low HDL-C	High WC
CC/AA	1.00	1.00	1.00	1.00	1.00
CC/AG	1.32 (0.83–2.11)	1.13 (0.63–2.06)	1.04 (0.60–1.78)	0.90 (0.56–1.43)	0.77 (0.48–1.23)
CC/GG	1.75 (0.72–4.28)	3.08 (1.21–7.82)	1.84 (0.72–4.71)	0.69 (0.28–1.70)	1.46 (0.58–3.66)
CT/AA	1.19 (0.83–1.69)	2.08 (1.35–3.21)	1.33 (0.89–1.98)	0.93 (0.66–1.32)	1.10 (0.77–1.57)
CT/AG	1.36 (0.93–1.98)	1.94 (1.22–3.09)	1.30 (0.84–2.00)	0.79 (0.54–1.16)	0.96 (0.65–1.41)
CT/GG	0.94 (0.47–1.88)	2.90 (1.37–6.12)	2.65 (1.30–5.41)	0.89 (0.45–1.76)	0.64 (0.32–1.31)
TT/AA	1.44 (0.98–2.11)	1.98 (1.24–3.17)	1.41 (0.92–2.18)	1.06 (0.72–1.56)	1.18 (0.79–1.75)
TT/AG	1.36 (0.88–2.10)	1.95 (1.16–3.26)	0.98 (0.59–1.62)	0.89 (0.58–1.37)	1.34 (0.86–2.09)
TT/GG	1.87 (0.95–2.04)	3.95 (1.93–8.05)	1.70 (0.83–3.49)	0.64 (0.32–1.28)	1.25 (0.63–2.51)

Abbreviations: *MTHFR*, methylenetetrahydrofolate reductase; *MTRR*, methionine synthase reductase; OR, odds ratio; CI, confidence interval; BP, blood pressure; FBG, fasting blood pressure; TG, triglycerides; HDL-C, high density lipoprotein cholesterol; WC, waist circumference.

**Table 8:** Adjusted odds ratios for combined *MTHFR* C677T and *MTRR* A66G genotypes according to the number of components of MetS.

Age and Sex Adjusted OR (95% CI)				
Combined Genotype	≥1 Components	≥2 Components	≥3 Components	≥4 Components
CC/AA	1.00	1.00	1.00	1.00
CC/AG	0.79 (0.43–1.44)	0.91 (0.57–1.45)	0.88 (0.54–1.45)	1.57 (0.76–3.25)
CC/GG	3.95 (0.51–30.65)	1.73 (0.66–4.50)	1.89 (0.78–4.58)	1.57 (0.42–5.86)
CT/AA	0.99 (0.61–1.60)	1.25 (0.87–1.79)	1.31 (0.91–1.88)	2.02 (1.15–3.54)
CT/AG	1.01 (0.60–1.70)	1.26 (0.85–1.86)	1.15 (0.78–1.71)	1.74 (0.95–3.18)
CT/GG	1.21 (0.46–3.17)	1.34 (0.66–2.74)	1.61 (0.81–3.24)	1.45 (0.50–4.21)
TT/AA	1.24 (0.72–2.13)	1.47 (0.99–2.21)	1.46 (0.98–2.16)	2.49 (1.38–4.49)
TT/AG	1.24 (0.68–2.29)	1.25 (0.80–1.96)	1.47 (0.94–2.29)	1.57 (0.80–3.11)
TT/GG	1.39 (0.50–3.86)	2.52 (1.15–5.51)	1.98 (1.01–3.89)	1.58 (0.58–4.31)

Abbreviations: *MTHFR*, methylenetetrahydrofolate reductase; *MTRR*, methionine synthase reductase; OR, odds ratio; CI, confidence interval.

## 3. Discussion

MetS is caused by the interplay of multiple genetic and environmental factors [6]. A growing body of evidence has linked HHcy with MetS and its individual components [7,8,9,10,11,12,13,34]. Although the precise mechanism by which HHcy promotes these disorders remains unclear, some investigators have proposed possibilities including promoting endothelial dysfunction, inducing insulin resistance, and affecting DNA methylation status [10,14,15,16,17]. The *MTHFR* C677T and *MTRR* A66G polymorphisms are important genetic risk factors for HHcy [18,21] and are considered to be associated with DNA hypomethylation, indicating that they may serve as potential determinants of MetS. To our knowledge, this is the first study to investigate the possible relationships of the *MTHFR* C677T and *MTRR* A66G polymorphisms with MetS among the Chinese Han population. We found that the *MTHFR* 677T allele carriers had an increased risk of MetS, and the 677TT genotype showed a significant correlation with high FBG, high WC, and having more MetS components, respectively. No association was observed between the *MTRR* A66G polymorphism and MetS. However, the 66GG genotype showed a significant association with high FBG and TG. Additionally, the MTRR A66G polymorphism may interact with the *MTHFR* 677TT genotype conferring a higher risk of MetS.

Many previous studies have investigated the relationships of the *MTHFR* C677T polymorphism with MetS and its individual components [22,23,24]. Three studies conducted respectively in type 2 diabetes, schizophrenia and colorectal cancer patients reported no relationship of the *MTHFR* C677T polymorphism with MetS [26,28,30]. On the contrary, Elligrond *et al.* [27] reported that schizophrenic patients carrying the 677T allele had an increased risk of MetS, and Kim *et al.* [29] observed that the 677T allele was associated with an increased risk of MetS compared to the 677C allele among Korean ischemic stroke patients. Additionally, two studies conducted, respectively, in Iran and in Greece, have investigated the relationship of the *MTHFR* C677T polymorphism with MetS among general population without severe diseases [24,35]. The *MTHFR* C677T polymorphism was found to be not significantly associated with MetS among the Iranian population [35], however, the 677T allele showed a 4.02-fold increased risk of MetS among the Greek population [24]. In agreement with the findings of Vasilopoulos *et al.* [24], Elligrond *et al.* [27] and Kim *et al.* [29], our results showed a strong association between the *MTHFR* 677T allele and MetS, suggesting that the allele might be a genetic risk factor for MetS in the Northern Chinese Han population. Many factors may contribute to the inconsistencies between our results and those reported by the aforementioned published studies. Firstly, genetic backgrounds, which can have an influence on disease susceptibility, are different among populations. Secondly, different populations have different dietary habits (such as intake of folate, vitamin B_2_ and vitamin B_12_) and environmental exposures, which may affect the association between the polymorphism and MetS through epigenetic mechanisms. Thirdly, some of these studies had relatively small sample sizes, which may lack sufficient power to deny or support an association. Fourthly, some studies are conducted in specific populations with various diseases like type 2 diabetes, schizophrenia, cancer and stroke, while others, including our study, were conducted among general population without any severe diseases, indicating that medication or other clinical factors might also have contributed to the inconsistencies. Lastly, differences in diagnostic criteria for MetS, mean age of the participants and sex proportion may also be responsible. In the analysis of the individual MetS components, the *MTHFR* 677TT genotype exhibited a significant association with high FBG, which is consistent with the results of previous studies [23,36]. These results could be explained by detrimental impact of Hcy on insulin secretion and pancreatic beta cell function [37,38]. Elevated Hcy levels caused by the *MTHFR* genetic variants have been demonstrated as related to insulin resistance, which is the major cause of high FBG or diabetes [39]. In addition, the correlation between the *MTHFR* 677T allele and DNA hypomethylation, which is suggested to be related to pathogenesis of diabetes, may be another possible explanation [15,19]. We also found a positive correlation between the *MTHFR* 677T allele and a higher likelihood of high WC. The link between the two has been investigated by several previous studies; however, the results were inconsistent [24,25,40]. Further studies therefore are still required to confirm or refute our findings.

The information about the effect of the *MTRR* A66G polymorphism on MetS and its individual components is limited. A recent study by Jiang *et al.* [41] reported that the 66GG genotype carriers had lower TC and LDL-C levels in comparison to those with the 66AA genotype in Chinese hypertensive patients. Another study of an Australian sample [42] found no association between the *MTRR* A66G polymorphism and the risk of hypertension. To our knowledge, this is the first study to evaluate the effect of the *MTRR* A66G polymorphism on susceptibility to MetS. Our study failed to find any association between the *MTRR* A66G polymorphism alone and the risk of MetS. However, it is noteworthy that the *MTRR* A66G polymorphism was associated with an increased risk of MetS when combined with the *MTHFR* 677TT genotype. Furthermore, our OR results evidently showed that the combined TT/GG, TT/AG and TT/AA genotypes conferred higher risk of MetS than the *MTHFR* C677T mutant genotypes alone. These data suggest that there might exist a synergistic effect of these two polymorphisms upon MetS causation. It was reported in several previous studies that the effect of the *MTRR* A66G polymorphism on plasma Hcy concentrations is weak [43], whereas coexistence of the *MTHFR* C677T and *MTRR* A66G polymorphisms was associated with an increased risk of HHcy compared to either the *MTHFR* C677T or *MTRR* A66G polymorphism alone [21,44]. Thus, it seems that the lack of correlation between the *MTRR* A66G polymorphism and MetS in our study may be due to its weak effect on plasma Hcy concentrations, and the synergistic effect of the two polymorphisms on MetS may be due to their interactive effect on Hcy concentrations. Analyses of the individual MetS components showed that the *MTRR* 66GG genotype was significantly associated with high FBG and TG. To the best of our knowledge, this is the first study reporting positive relationships of the *MTRR* A66G polymorphism with high FBG and TG. However, these results should be interpreted with great caution because the frequency of the *MTRR* 66GG genotype is relatively lower in our study. Therefore, further studies with larger sample sizes will be needed to confirm these findings.

In interpreting the findings of our study, several limitations should be acknowledged. Firstly, the study subjects were from one hospital, indicating that they may not be fully representative of the general population. Moreover, only two polymorphisms were explored for their associations with MetS, not all of the polymorphisms reported in the literature, indicating that gene-gene interactions may not be fully explored. Thirdly, information on environmental factors such as diet or behavior was not available, which suggests the effect of gene-environmental interactions may not be identified in the study. Despite the limitations, our study still has several strengths: (1) exploring the relationships of the *MTHFR* C677T and *MTRR* A66G polymorphisms with MetS for the first time in a Chinese population; (2) having large sample sizes, thus ensuring enough power to reduce type І error; (3) selecting ethnically homogeneous population (Chinese Han), thus improving the validity of statistical analysis.

## 4. Experimental Section

### 4.1. Ethics Statement

The study was conducted in accordance with the principles stipulated by the Declaration of Helsinki and all procedures were approved by the ethics review committee of China Medical University (Shenyang, China; Identification code: CMU62073024; 15 July 2008). All specimens and survey data were obtained with written informed consent from all participants prior to study entry and subsequently anonymised.

### 4.2. Study Subjects

Between October 2008 and February 2011, a total of 692 MetS patients and 878 healthy controls who took regular physical examination at the physical examination center of Dagang Oil Field General Hospital were recruited into our study. Patients and controls were frequency-matched according to age (±5 years) and sex. All subjects were genetically unrelated ethnic Han Chinese and resided in the Dagang district of Tianjin municipality, northern China. Main exclusion criteria included a history of myocardial infarction, stroke, diabetes, any type of cancer, and renal failure or chronic liver disease. Weight, height and WC were measured using a standard scale with light clothing and barefoot after an overnight fast. At the same time, participants were asked for permission to store a blood sample for biochemical analysis and a buccal cell sample for genetic analysis. BMI was calculated as weight in kilograms divided by the square of height in meters (kg/m^2^). BP was measured while subjects were in the sitting position after 15 min of rest. The average of three measurements was recorded.

### 4.3. Biomedical Measurements

The levels of TC, HDL-C, LDL-C, TG and FBG in samples were determined by enzymatic method using a Hitachi Autoanalyzer (Type 7170A; Hitachi Ltd., Tokyo, Japan) in Dagang Oil Field General Hospital. All assays were performed according to the manufacturer’s instructions.

### 4.4. Definition of the MetS

MetS was defined using the Updated National Cholesterol Education Program/Adult Treatment Panel III Criteria for Asian Americans as having at least three of the following components: (1) BP of at least 130/85 mmHg and/or on antihypertensive medications; (2) TG of at least 1.7 mmol/L; (3) HDL-C less than 1.03 mmol/L for males and less than 1.30 mmol/L for females; (4) FBG of at least 5.6 mmol/L or taking medication for diabetes mellitus; (5) WC greater than 90 cm for males and greater than 80 cm for females [45].

### 4.5. Genotyping

Genomic DNA was extracted from buccal samples using the QIAamp DNA Mini Kit (Qiagen, Valencia, CA, USA). Genotyping for the *MTHFR* C677T and *MTRR* A66G polymorphisms was performed as described in our previous paper [33].

### 4.6. Statistical Analysis

The *MTHFR* C677T and *MTRR* A66G allele and genotype frequencies in the patients and controls were calculated by direct counting. χ^2^ analysis was performed to identify departure from the Hardy–Weinberg equilibrium, and to compare the differences between the two groups with respect to allelic and genotypic frequencies. The unconditional logistic regression analysis was performed to estimate the effects of the *MTHFR* C677T and *MTRR* A66G polymorphisms singly and in combination on MetS and its individual components after adjustment for age and sex. The OR with 95% CI was calculated to estimate the relative risk of the different genotypes and alleles. A *p* value below 0.05 was taken as statistically significant. All analyses were performed using SAS version 9.2 (SAS Institute, Cary, NC, USA).

## 5. Conclusions

In conclusion, in this study we found a significant association between the *MTHFR* 677T allele and MetS, high FBG, high WC, and increasing number of MetS components in a Chinese Han population. The *MTRR* A66G polymorphism was not associated with MetS. However, there might exist an interactive effect of the *MTHFR* C677T and *MTRR* A66G polymorphisms on MetS. Furthermore, the *MTRR* 66GG genotype showed a significant association with high FBG and TG. These findings are of significance, as these are valuable for the early identification of individuals at high risk for MetS in later life and for motivating them to adopt a healthy lifestyle (such as folic acid fortification) to reduce the risk. However, as in any study examining genotype-phenotype relationships, replication of the current study is required. Therefore, further large prospective population-based studies are required to confirm our findings.
